# Data-efficient prediction of OLED optical properties enabled by transfer learning

**DOI:** 10.1515/nanoph-2024-0505

**Published:** 2025-02-10

**Authors:** Jeong Min Shin, Sanmun Kim, Sergey G. Menabde, Sehong Park, In-Goo Lee, Injue Kim, Min Seok Jang

**Affiliations:** School of Electrical Engineering, Korea Advanced Institute of Science and Technology, Daejeon, South Korea; Division of Physics, Mathematics, and Astronomy, California Institute of Technology, Pasadena, CA 91125, USA; CTO Division, LG Display Co., Seoul, 07796, Republic of Korea

**Keywords:** organic light-emitting diode, light extraction efficiency, transfer learning, machine learning

## Abstract

It has long been desired to enable global structural optimization of organic light-emitting diodes (OLEDs) for maximal light extraction. The most critical obstacles to achieving this goal are time-consuming optical simulations and discrepancies between simulation and experiment. In this work, by leveraging transfer learning, we demonstrate that fast and reliable prediction of OLED optical properties is possible with several times higher data efficiency compared to previously demonstrated surrogate solvers based on artificial neural networks. Once a neural network is trained for a base OLED structure, it can be transferred to predict the properties of modified structures with additional layers with a relatively small number of additional training samples. Moreover, we demonstrate that, with only a few tenths of experimental data sets, a neural network can be trained to accurately predict experimental measurements of OLEDs, which often differ from simulation results due to fabrication and measurement errors. This is enabled by transferring a pre-trained network, built with a large amount of simulated data, to a new network capable of correcting systematic errors in experiment. Our work proposes a practical approach to designing and optimizing OLED structures with a large number of design parameters to achieve high optical efficiency.

## 1 Introduction

Organic light-emitting diodes (OLED) is a mainstream display technology adopted in a wide range of devices spanning from wearable and mobile devices to large-screen televisions and signages. Both electrical and optical properties of OLED have been constantly improved [1], with a special focus on the external quantum efficiency (EQE) [2], [3], [4], [5], which is a ratio between the number of photons emitted by the device and the number of electrons injected into the device. The EQE is given by the product of the light extraction efficiency (LEE) and the internal quantum efficiency (IQE). With the development of light emitting organic molecules, IQE has reached near unity [6], [7], thus the LEE has become the limiting factor dictating the overall device quantum efficiency [8]. Consequently, improving the LEE by structure optimization has been the subject of many previous studies [9], [10], [11].

Various electromagnetic simulation techniques have been developed and adopted to numerically design the LEE of OLEDs. Conventional full-wave simulations based on the finite-difference time-domain (FDTD) and finite element methods (FEM) provide general and convenient ways to predict optical properties of OLEDs but they are computationally expensive. The computational complexity can be greatly reduced when the device structure possesses a strong symmetry. For example, Chance, Prock, and Silbey developed a model (called CPS model) that analyses the interaction between fluorescent molecules and the nearby metal surface, and enables the analysis of the near field radiation of the emissive layer in planar OLEDs to calculate the light extraction efficiency of the device [12]. This method was later extended to periodically corrugated devices by Park et al. [13] Although these two approaches have alleviated computational burden to some degree and thus enabled optimizations of simple devices with a small number of design variables, they are still not fast enough for global optimization of more general structures with a large parametric space and multiple constraints such as color coordinates and viewing angle, which are crucial for meeting industrial demands.

To address this issue, machine learning has been used to boost the computation speed by using artificial neural networks that produce approximate results rather than exact solutions. Once trained, surrogate solvers based on artificial neural networks predict the optical properties of the devices a few orders of magnitude faster than the rigorous simulations and therefore can tackle the high-complexity optimization problems [14]. However, there exist critical challenges to overcome. First, a large amount of training data, which needs to be generated by rigorous simulations, is required to train a network with sufficient accuracy [15]. Second, even a slight change in the device configuration (e.g., new layers are added) requires the entire network to be retrained with a new set of training samples, which, again, involves time-consuming sample generation via rigorous electromagnetic simulations. Finally, since the network is trained by the simulated data, theory versus experiment discrepancies are inevitable due to the fabrication, material properties, or measurement errors, negatively affecting the performance of fabricated devices designed by a neural network.

In this study, we suggest a pathway to overcome the aforementioned problems by using the transfer learning – a deep learning method based on the migration of certain deep layers from a pre-trained network [16], [17]. This approach allows for efficient network learning even when the training situation changes as reported several times for different optical devices [18], [19], [20], [21]. We apply transfer learning to predict light emitting properties of OLEDs and demonstrate a two-fold enhancement of data efficiency compared to the case of direct learning. Moreover, we show that transfer learning can also be leveraged to lift the simulation-experiment mismatch problem with only a few dozens of experimental measurement data sets.

## 2 Transfer learning for light extraction efficiency prediction in OLED

**Figure 1:**
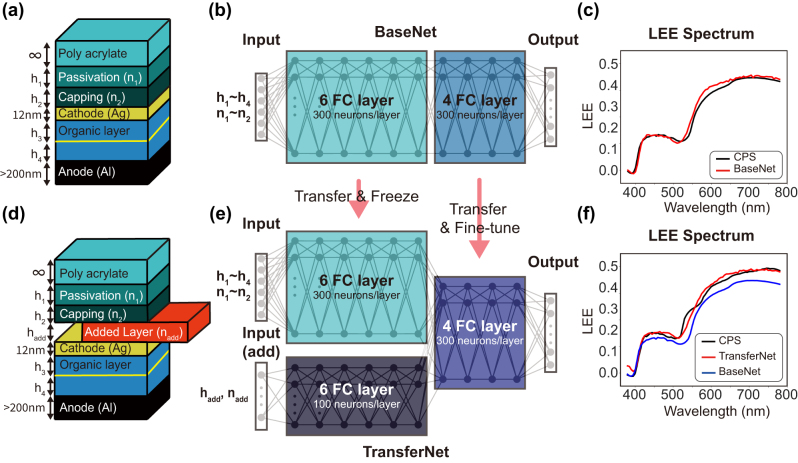
Schematic of LEE prediction network with transfer learning. (a) and (d) Show the 6-design-variable OLED and 8-design-variable OLED, respectively. (b) and (e) Illustrate the LEE prediction networks for 6- and 8-variables OLEDs: the BaseNet and the TransferNet, respectively. The BaseNet is transferred to the TransferNet in two parts: frozen weight parameters (cyan) and tunable (blue) weight parameters. (c) and (f) Show the LEE spectra predicted by the BaseNet and the TransferNet, respectively, including the CPS-calculated LEE spectra (black), used as a ground truth. The blue spectrum in (f) shows the LEE spectrum of the 8-parameter OLED calculated by the BaseNet with 6 parameters ignoring the 2 addtional parameters for the added layer, as shown in (b).

The considered OLED structure is a multilayer stack illustrated in Figure 1(a). The organic diode layer is N,N′-Di(1-naphthyl)-N,N′-diphenyl-(1,1′-biphenyl)-4,4′-diamine (NPB) [22], which is located above the aluminum (Al) anode. To maintain the electrical properties of the cathode and anode layers, their thickness is fixed at 12 nm and 100 nm, respectively.

The training samples for neural networks in our study are generated by rigorous simulations using the CPS model under the assumption of non-absorbing emitting medium, isotropic dipole moment transition, low excitation level, and relatively small excitation zone compared to the cavity length. Such CPS model approximations have been shown to provide a good agreement with experimental results in OLEDs [23], [24], [25], [26], [27], hence we apply the same model to calculate the LEE in this work.

The possible range of the refractive indices of the passivation (*n*_1_) and capping (*n*_2_) layers is 1.6–1.9 and 1.4 to 2.0, respectively, and the thickness of the layers can vary between 500 nm and 1,500 nm for the passivation layer (*h*_1_) and 10 nm and 250 nm for the capping layer (*h*_2_) [14]. Both distances from the light-emitting layer to the top (*h*_3_) and bottom (*h*_4_) boundaries of the NPB layer are in the range of 10 nm and 250 nm, respectively. These six specific parameters can change the optical properties without significantly affecting the electrical characteristics of the device. The dipolar emission from the emission layer is assumed to be randomly oriented. For every given structure, LEE is calculated at 81 wavelengths between 380 nm and 780 nm with a step of 5 nm. Hence, each training sample has 6 structure parameters (*h*_1_, *h*_2_, *h*_3_, *h*_4_, *n*_1_, *n*_2_) as input and the LEE spectrum at 81 wavelengths (380, 385, 390, … , 780 nm) as output.

As a first step, we construct and train the network called BaseNet to predicts the LEE spectrum of the OLED device with 6 design parameters discussed above (Figure 1(b)). The BaseNet consists of 10 fully connected layers with 300 neurons in each layer and is trained with the Adam optimizer on 2000 training samples to minimize the root mean squared error (RMSE) between the predicted LEE spectra and the ground truth spectra. This training sample size is significantly smaller compared similar studies where 260,000, 750,000 samples are used [14], [28]. The model is subsequently trained on the previous 2000 training sample using a batch size of 500 across 2000 iterations. The RMSE of the LEE spectrum predicted by the BaseNet is 0.0168 (calculated from 1,000 test samples). Once trained, the neural network performs feedforward operations on a given input to produce a predicted LEE spectrum, and this process is called inference. The comparison of a rigorously simulated LEE spectrum with the BaseNet prediction for the same device is shown in Figure 1(c). We note that the CPS model run on a single core CPU (SPECIFY CPU model) requires 23 s to compute the LEE of a given OLED structure, while the networks in this paper takes only 0.53 ms on the same CPU, showing a four orders of magnitude faster calculation. Moreover, BaseNet calculation time dramatically reduces to 0.08 μs per structure with NVIDIA^®^ GeForce RTX™ 3080 GPU.

We then create another network, the TransferNet, that predicts the LEE spectrum of OLED structures with one additional layer that adds two new design parameters into the system: thickness (*h*_add_) and refractive index (*n*_add_). Thus, TransferNet has 8 design parameters as inputs – the original 6 plus *n*_add_ and *h*_add_. The additional layer is located between the cathode and the capping layer as shown in Figure 1(d), and is expected to have a large impact on the LEE spectrum while not affecting the electrical properties of the structure. To consider various materials, the *n*_add_ is allowed to vary from 1.2 to 2.0, and its *h*_add_ is in the range from 10 nm to 1,000 nm.

The TransferNet consists of three parts. The first part is transferred from the first *M* layers of the BaseNet and “frozen” parameters weights (indicated as cyan box in Figure 1(e) for when *M* = 6). The second part is a new network that takes additional parameters as an input, and the third part connects the two previous networks, which are transferred from the last (10 − *M*) layers of the BaseNet, but with un-frozen weight parameters (indicated as purple box in Figure 1(e)). Then, the TransferNet is trained on training samples of different sizes *N* between 200 and 2000 in increments of 200.

To verify the accuracy of BaseNet and TransferNet, Figure 1(f) compares the LEE spectra of the new OLED structure with 8 design parameters calculated by the CPS model (black) with the LEE predicted by the TransferNet with (*N*, *M*) = (1,000, 6) (red) and by the BaseNet (blue). The TransferNet shows much better agreement with the ground truth compared to the prediction results by the BaseNet which ignores the added layer.

**Figure 2:**
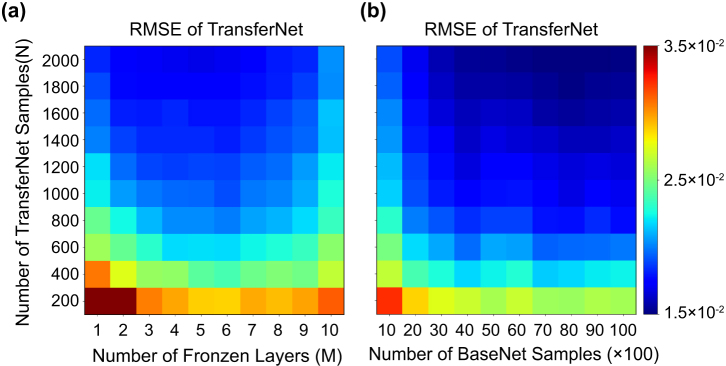
The RMSE of the TransferNet as a function of (a) the number of TransferNet training samples (*N*) and the number of frozen layers (*M*), with the BaseNet trained on 2,000 samples; and (b) the number of TransferNet training samples (*N*) and the number of BaseNet training samples, with a fixed number of frozen layers (*M* = 6).

When the number of training samples for the BaseNet is fixed at 2000, the performance (RMSE value) of the TransferNet depends on the number of frozen layers transferred from the BaseNet (*M*) and the number of training samples for the TransferNet (*N*) as shown in Figure 2(a). The RMSE of the TransferNet monotonically decreases as more samples are used for its training. Interestingly, the TransferNet shows the best performance for *M* = 6 regardless of *N*. The existence of an optimal number of frozen layers in the transferred network suggests that too few frozen layers cannot carry sufficient information from the pre-trained network, while too many frozen layers cause the network to be less adaptive to the new OLED structure. Therefore, choosing an appropriate number of fixed layers determines the overall performance of the network.

The performance of the TransferNet also depends on the accuracy of the BaseNet. Figure 2(b) shows the relationship between the RMSE of the TransferNet and the size of the training sets for both the TransferNet and the BaseNet for *M* = 6. As expected, as the size of both training sets increases, the prediction error decreases. At the same time, the number of samples for the BaseNet training has limited effect as the RMSE saturates after the set size exceeds approximately 2,000 samples. This suggests that there exists a limit to how much the TransferNet performance can be improved by the BaseNet training. The TransferNet performance also saturates as the size of its own training set increases, which is likely due to the existence of the frozen layers because they contain the error of the pre-trained network. What is remarkable, however, is that only several hundred samples are sufficient to obtain RMSE value less than 0.02 using the TransferNet. Compared to previous research that apply the transfer learning in photonics, transfer learning in this paper dramatically improves the accuracy even with relatively inaccurate BaseNet [20], [21].

**Figure 3:**
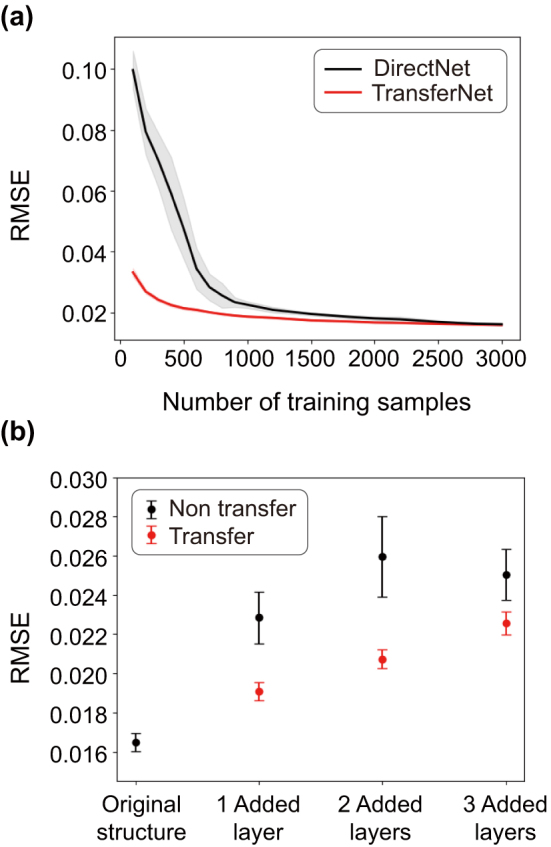
Average prediction error of TransferNet and DirectNet. (a) The average RMSE of TransferNet (red), which uses the BaseNet trained on 2,000 training samples and the average RMSE of DirectNet (black). Both networks were trained on, and the resulting RMSE values were averaged over, 30 independent training sample sets. The shaded area represents the standard deviation. (b) The RMSE of TransferNet and DirectNet with 1, 2, and 3 additional layers in the OLED structure, averaged across 40 independent training sample sets containing 1,000 training samples each. Error bars indicate the standard deviation.

The effectiveness of transfer learning can be estimated by comparing the TransferNet performance with that of a network with the same architecture, but trained on an independent set of samples starting from randomly initialized weights without transfer learning, which is referred to as the DirectNet. To make the statistical analysis, we analyze 30 networks trained by different sets of 2000 training samples and show the mean value and the standard deviation of RMSE obtained from both networks in Figure 3(a). Notably, TransferNet shows a standard deviation of 4.6 × 10^−4^ across 500 training data samples, while DirectNet shows an order of magnitude larger standard deviation of 9.9 × 10^−3^. It can also be seen that transfer learning increases data efficiency by more than 200 % which is very high compared to other studies using transfer learning [19], as DirectNet requires more than 1,000 training samples to reach the prediction accuracy (RMSE = 0.0214) of TransferNet on 500 training data samples. This means that the transfer learning not only makes the network more stable against the training set changes, but also results in much better data efficiency which is particularly prominent at smaller training sets. However, the performance difference between the two networks decreases as the number of training samples (*N*) increases, and becomes negligible when it surpasses the number of samples used to train the BaseNet.

We proceed with testing the effectiveness of transfer learning when the OLED structure becomes even more complex. While the discussion above has been focused on the LEE prediction in the OLED with a single additional layer, we also statistically analyze the effectiveness of transfer learning when 2 and 3 layers are added to the original OLED structure. Again, we compare the performance of the TransferNet with (*N, M*) = (1,000, 6) and the DirectNet (*N* = 1,000) without transfer learning. The number of input parameters increases from 6 in the original OLED up to 8, 10, and 12 in the OLED with 1, 2, and 3 extra layers, respectively. All three new TransferNets are trained with different training sets, and their performance is analyzed by predicting the LEE for 1,000 test structures for each case. To validate the robustness of the network against the BaseNet and training data, we compare the LEE prediction accuracy of 40 different TransferNets trained using different training data sets, while every BaseNet is also trained using an independent dataset of 2000 samples. As shown in Figure 3(b), the TransferNet shows 10–25 % lower RMSE compared to the DirectNet with the same 1,000 training samples. The standard deviation of RMSE, which shows the stability of the network, is also 2.3–4.2 times smaller for the TransferNet. This suggests that transfer learning improves network performance even in the case of more complex structures, which are significantly different from the original OLED structure. We also demonstrate that the TransferNet can be used to optimize 8-layer OLED structure more than 10^3^ times faster than the rigorous CPS model, but the results of the two optimizations were very similar, as shown in Figure S1. In addition, because TransferNet is more than twice as data-efficient, the time to train the network is also more than twice as fast as the existing network. This suggests that the TransferNet could allow for a faster OLED structure optimization compared to not only computational models but also existing machine learning networks.

## 3 Transfer learning for error prediction

In many cases, experimentally realized devices exhibit different properties compared to those predicted by numerical simulations [29]. This discrepancy can be attributed to various sources of error. Here, we classify the errors into two categories: systematic and random errors. As a representative example of systematic errors, we consider a systematic deviation of the design parameters of a fabricated device from their design values, often caused by miscalibrated fabrication equipment. We also consider random errors in optical measurements due to unidentifiable sources such as detector noise.

**Figure 4:**
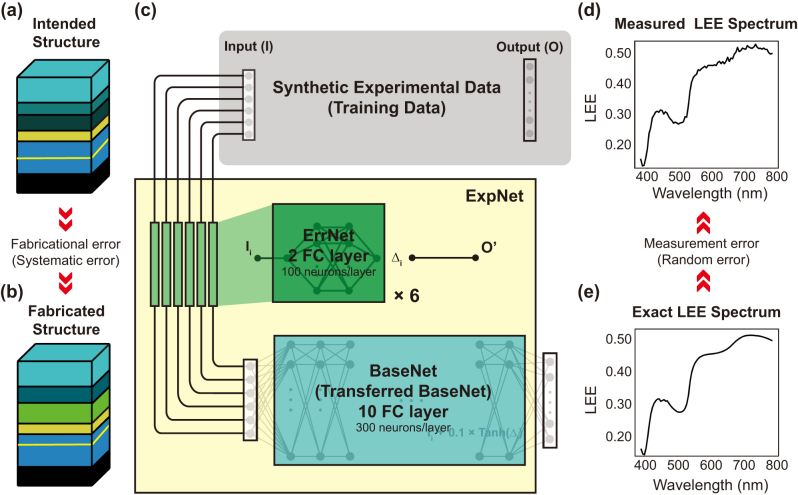
Schematic of the error prediction network. (a), (b) The intended and fabricated OLED structure sample, respectively. The intended structure is identical to the condition shown in Figure 1. (a) While the fabricated structure includes fabrication errors in both the refractive index and thickness. (c) The error prediction network structure for the experimental dataset (gray box), which accounts for both systematic and random errors. The ExpNet (yellow box) consists of the transferred and frozen BaseNet (blue box) and the ErrNet (green box) which has two hidden layers, each containing 100 neurons. (d), (e) The LEE spectra calculated from the fabricated structure with and without random measurement error, respectively.

In this work, we suggest a way to predict the measured LEE spectra from the intended device structures using extremely small amount of experimental data in combination with a large amount of simulation data. This approach is meaningful because it usually takes significantly more time and effort to obtain experimental measurement data compared to running an optical simulation. Due to the systematic fabrication errors, the intended device structures, Figure 4(a), can be different from the actual fabricated ones, Figure 4(b). Furthermore, random noise makes the measured LEE spectra, Figure 4(d), different from the calculated exact spectra, Figure 4(e). Since the random measurement error is inherently unpredictable, here we focus on analyzing and correcting the systematic errors using transfer learning.

To mimic experimental measurements, we generate synthetic experimental data with artificial errors as illustrated in Figure 4. We apply systematic error functions for each input design parameter within 10 % of its design value (see Supplementary 02 for more details), and random Gaussian error is assigned to the output LEE spectra. This synthetic experimental data allows us to verify whether the network can correctly predict the known systematic error function.

A new network called ExpNet is constructed by prepending an additional network (called ErrNet) to the pre-trained and fully transferred BaseNet, which has the same network structure as the one used in the previous sections but trained with 50,000 samples, as shown in Figure 4(c). The ErrNet is responsible for predicting the systematic fabrication errors in the device structure. It takes intended design parameters as an input and produces the parameters of the fabricated structure that contains the systematic errors that occurred during fabrication as shown in Figure 4(a and b). Because of the assumed systematic error function is a function of that parameter alone, we use six different ErrNets for six input design parameters and each ErrNet is composed of two fully connected hidden layers with 100 nodes each as indicated as the green box in Figure 4(c) (see Supplementary 02 for more details).

**Figure 5:**
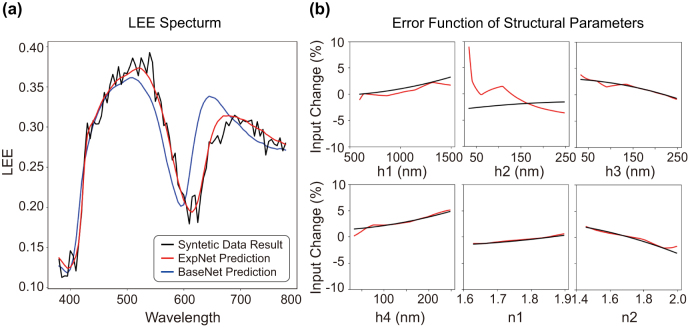
Performance of ExpNet and ErrNet. (a) The LEE spectrum of the synthetic experimental data containing both systematic and random errors (black), and the predicted LEE spectra by the ExpNet (red) and the BaseNet (blue). (b) Input systematic error functions for the design parameters (black) and the predicted error functions by the ErrNet (red).

During the training of the ExpNet on the experimental data, the pre-trained and transferred BaseNet is frozen and only the ErrNets are trained. Since the LEE prediction accuracy of the BaseNet constrains the overall prediction accuracy of the ExpNet and thus affects the error correction capability of the ErrNet, we first train the BaseNet with a much larger amount of data (50,000 samples) compared to the previous case. The ExpNet is then trained using only 60 synthetic experimental samples containing both systematic input errors and random output errors with standard deviation of 0.01. Figure 5(a) shows an example of the resulting LEE spectra predicted by the ExpNet (red) and the BaseNet (blue) together with the synthetic experimental data (black). The ExpNet prediction agrees well with the synthetic experimental data, except for the Gaussian random noise. Indeed, the RMSE of the trained ExpNet reaches down to 0.0112, close to the noise floor defined by the random Gaussian error. In contrast, when the systematic error is ignored (ErrNets are turned off), the RMSE of the BaseNet for synthetic experimental data is 0.1, an order of magnitude higher than that of ExpNet, demonstrating the effectiveness of transfer learning.

Even with random Gaussian errors in the output LEE spectra, the ErrNet is able to identify the assigned systematic error functions of input parameters as shown in Figure 5(b) (see Figure S3 for more details). We note that, although the ExpNet can accurately predict the synthetic experimental data, the ErrNet prediction for the parameter *h*_2_ (the capping layer thickness) shows poor prediction accuracy. This can be explained by the different impact of each design parameter on LEE and the limited effect of h2 on the LEE in the considered OLED structure (see Figures S4 and S5) [30]. Thus, the accuracy of the error prediction function can also be used to qualitatively estimate the effect of a given variable on the LEE spectrum.

**Figure 6:**
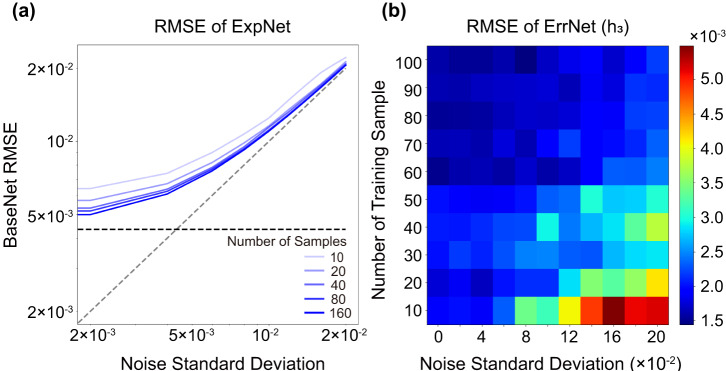
The RMSE of ExpNet and ErrNet as a function of the noise level and the number of training samples. (a) The RMSE of the ExpNet trained on 10–160 samples as a function of the standard deviation of the random noise in the synthetic experimental LEE and that of the BaseNet. The gray and black dotted lines represent the standard deviation of the random noise and the BaseNet RMSE, respectively. (b) The RMSE of the ErrNet for the parameter *h*_3_ as a function of the number of training samples and the standard deviation of the random noise.

The RMSE of ExpNet is inherently bounded by the noise floor and the RMSE of the BaseNet. Figure 6(a) shows the average RMSE of the ExpNet for 10 different systematic error functions in the synthetic data as a function of the number of training sets, as well as the theoretical limit for RMSE given by the pre-trained BaseNet (black dashed) and the Gaussian random noise (gray dashed). Notably, the ExpNet approaches the theoretical limit even when only a few dozen samples are used for its training. Additionally, Figure 6(a) shows that the prediction accuracy of the ExpNet saturates as the training sample size increases. At the same time, since the number of training samples is very small, it shows the effectiveness of transfer learning in the analysis of experimental data. However, as the output random error (Gaussian noise) increases, RMSE saturates to its standard deviation because random noise surpasses the network prediction error. In this regime, it is difficult to evaluate the performance of the network based on the RMSE alone. Therefore, we proceed with the separate analysis of ErrNet performance.

The performance of the ErrNet, which is critical for the ExpNet, can be evaluated by the accuracy of the network’s predictions with respect to the number of training samples and the robustness of the network to random errors. For example, RMSE of the ErrNet’s prediction for parameter *h*_3_ is shown in Figure 6(b) as a function of the number of training samples and the standard deviation of the Gaussian random error. RMSE is statistically calculated based on 10 different error functions for *h*_3_. The RMSE of ErrNet generally increases with the standard deviation of the random noise. In particular, at a high noise level with the standard deviation over 0.01, the network prediction error tends to blow up when it is trained on too few samples. To prevent this error blow-up, one needs 60 or more experimental data sets for network training, but this is still a sufficiently small amount that can be generated by actual experiments. The ErrNets for other input design parameters show similar dependence on the number of training samples and the noise level, which are presented in Figure S5.

## 4 Conclusion and discussion

In summary, we leverage transfer learning to tackle two prominent problems in predicting optical properties of OLEDs. First, with transfer learning, a neural network that is trained for a certain OLED structure can be reused for modified structures having additional layers, resulting in a two-fold increase in sample efficiency compared to the case of direct learning with the same network architecture. Second, we show that the long-standing problem of simulation-experiment mismatch can also be addressed with transfer learning. By combining an error correction network with an accurate surrogate solver trained with a larger amount of simulation data, the whole combined network can be trained to predict experimental LEE spectra by using only a few dozens of experimental training samples. We note that the experimental data used in this work are synthetically generated to prove the concept. To further validate the practical effectiveness of the proposed approach, it is necessary to apply it with real experimental data. Our work constitutes a stepping stone towards a global structural optimization of OLED structure by enabling a fast and reliable prediction of OLED optical properties.

## Supplementary Material

Supplementary Material Details
