# Supplementary material for: Data-efficient prediction of OLED optical properties enabled by transfer learning
Source: Nanophotonics. 2025 Feb 10;14(8):1091–9. doi: 10.1515/nanoph-2024-0505 (PMC12019943; doi:10.1515/nanoph-2024-0505)
Supplement: Supplementary file 1 — Supplementary Material Details [file j_nanoph-2024-0505_suppl_001.docx]

**Supplementary Information**

**Data-efficient prediction of OLED optical properties enabled by transfer learning**

Jeong Min Shin^1^, Sanmun Kim^1, 2^, Sergey G. Menabde^1^, Sehong Park^3^, In-Goo Lee^4^, Injue Kim^4^ and Min Seok Jang^1, *^

^1^ School of Electrical Engineering, Korea Advanced Institute of Science and Technology, Daejeon 34141, Republic of Korea

^2^ Division of Physics, Mathematics, and Astronomy, California Institute of Technology, Pasadena, CA 91125

^3^ OC Optical Technology Task, LG Display, Seoul, 07796, Republic of Korea

^4^ CTO Division, LG Display Co., Seoul, 07796, Republic of Korea

* Email: [jang.minseok@kaist.ac.kr](mailto:jang.minseok@kaist.ac.kr)

**Supplementary section 01**

OLED optimization results by a genetic algorithm with Transfer learning that maximizes the average LEE value in the visible wavelength range (380 nm – 780 nm) are shown in **Fig. S01**. Optimization process takes 100 “populations” and 50 iterations. Compared to the optimization based on the CPS model (red in **Fig. S01**), the optimization flow with transfer learning (black in **Fig. S01**) suffers setbacks at certain iterations, which is not typical for a genetic algorithm optimization. The reason for such setbacks is that the optimization is based on a predicted LEE rather than the exactly computed values, so when the computed values are obtained, there will be iterations where the LEE value decreases. The optimized average LEE from Transfer learning and CPS are 0.424 and 0.425, respectively. The optimized structural parameter vector [$h_{1}, h_{2}, h_{add}, h_{3}, h_{4}, n_{1}, n_{2}, n_{\mathrm{add}}$] = [967.8 nm, 109.7 nm, 95.7 nm, 76.4 nm, 30.8 nm, 1.752, 1.547, 2.0] and [942.8 nm, 52.2 nm, 95.17 nm, 81.29 nm, 20.9 nm, 1.862, 1.523, 2.0] for Transfer learning and CPS, respectively.


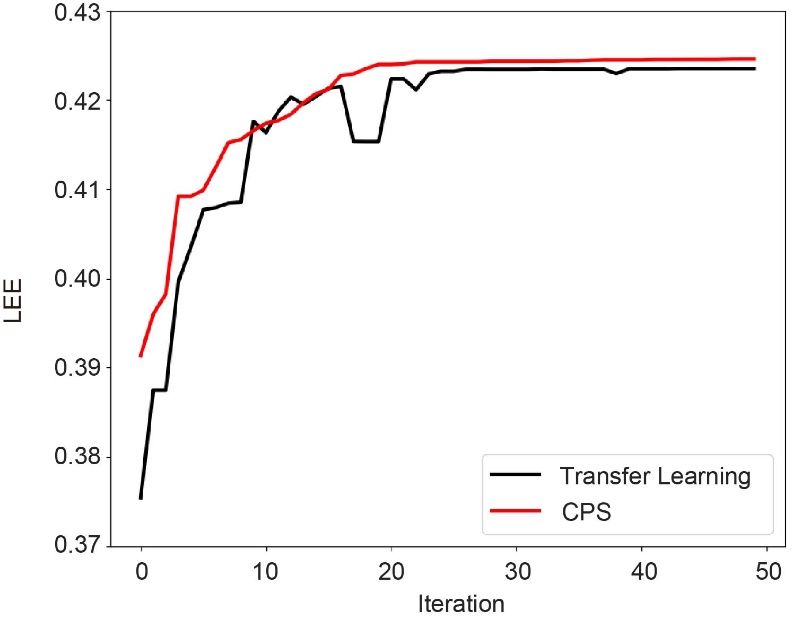


**Supplementary Figure S01.** LEE optimization curve with genetic algorithm from the transfer learning (black) and CPS (red) respectively.

**Supplementary section 02**

Error function is given by: $y={ax}^{2}+bx+c$, where $a, b, c$ are randomly chosen such that they satisfy $\left| a \right|, \left| c \right|<\frac{0.1}{3}$ and $1-\frac{0.1}{3}<b<1+\frac{0.1}{3}$ to make upper and lower boundaries of the error to be 0.1 of the parameter value. In addition, random error with a Gaussian distribution is added to the output data to simulate the measurement error due to noise. To predict the systematic error *y*, which is assumed to be around 10%, the output parameter value of ErrNet is obtained by taking the hyperbolic tangent of the error value, multiplying it by 0.1, and adding it to the original parameter value.

**Supplementary section 03**

Both **Fig. S02** and **Table S01** show the structural parameters of an arbitrary structure. In this structure, ErrNet is highly predictive of functional errors in structural parameters except for h2. The reason ErrNet cannot predict the error of h2 is that h2 does not have a significant impact on the LEE spectrum.


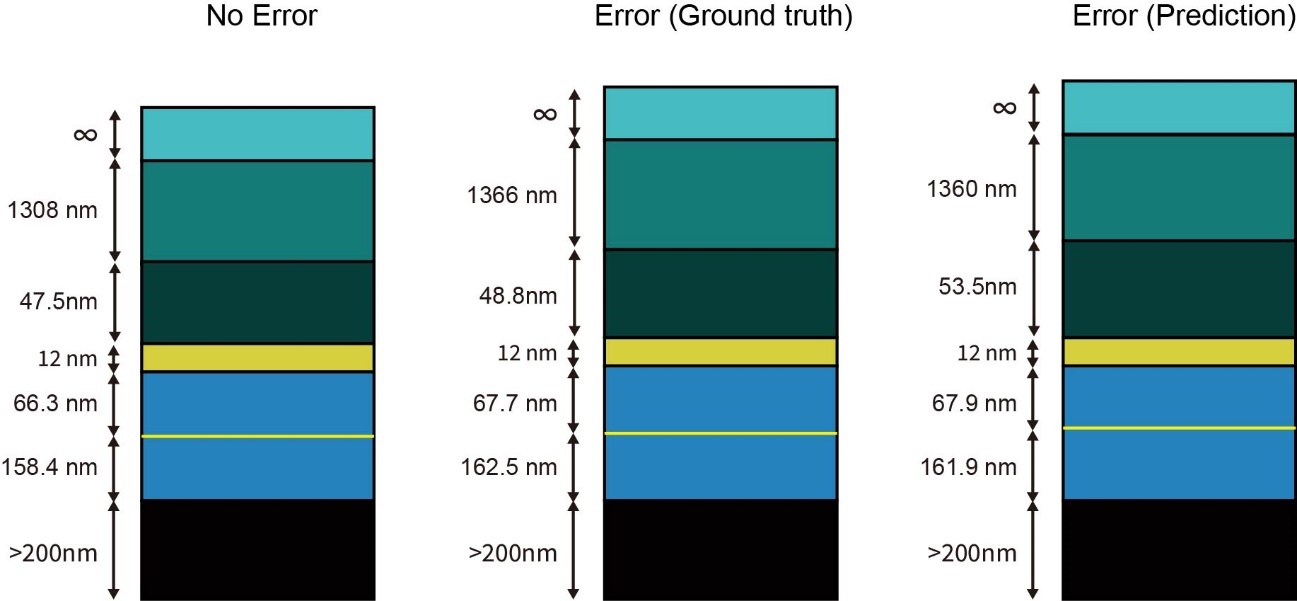


**Supplementary Figure S02.** Structure parameters of arbitrary OLED without systematic error (left), with systematic error (middle), and predicted systematic error (right).

|  | **No Error** | **Error Ground Truth** | **Error Prediction** | **Prediction Accuracy** |
| --- | --- | --- | --- | --- |
| h1 | 1308 nm | 1366 nm | 1360 nm | -0.0046 |
| h2 | 47.49 nm | 48.76 nm | 53.46 nm | 0.0964 |
| h3 | 66.28 nm | 67.74 nm | 67.88 nm | 0.0020 |
| h4 | 158.4 nm | 162.5 nm | 161.9 nm | -0.0035 |
| n1 | 1.662 | 1.682 | 1.684 | 0.0012 |
| n2 | 1.485 | 1.508 | 1.519 | 0.0079 |

**Supplementary Table S01.** Structure parameter of arbitrary sample without systematic error, with systematic error and predicted systematic error, respectively.

**Supplementary section 04**

The 5% deviation for every parameter is assigned to check for the sensitivity of LEE to each parameter. Unlike the other variables, h2 does not have a significant effect on the LEE (**Fig. S03**). This leads to the poor prediction accuracy for h2 from LEE spectra for the considered OLED structure. Consequently, only h2 shows a high prediction error provided by ErrNet.


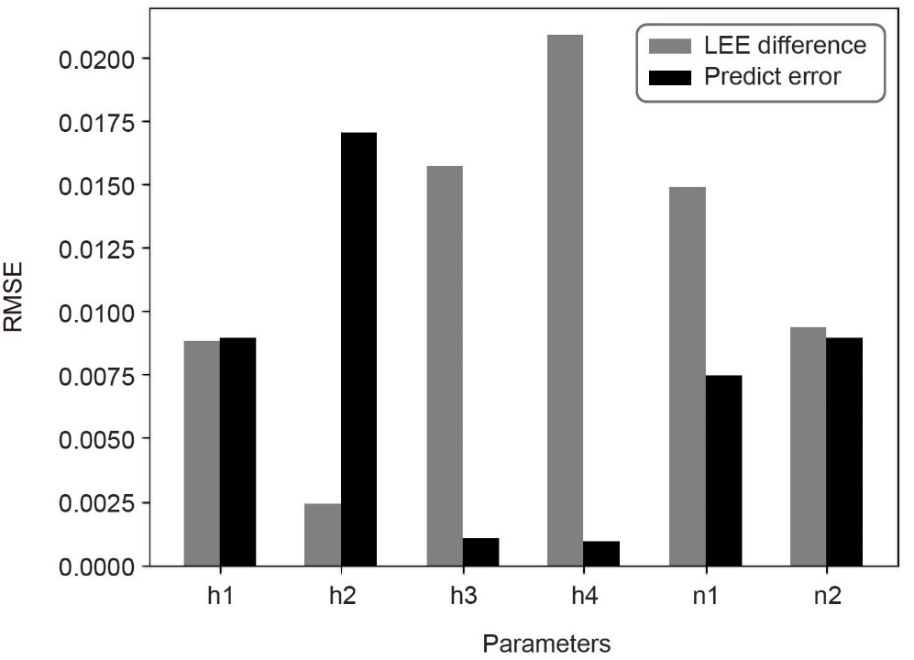


**Supplementary Figure S03.** RMSE of the error prediction for each structural parameter (black) and the LEE difference due to the perturbation of this parameter (gray).

**Supplementary section 05**

We compare the LEE spectrum of the synthetic experimental data with the calculated LEE spectrum from the predicted systematic error. Input structural data of each figure is shown in the **Table S02**. Although the LEE difference exceeds 10% for the structural parameter h2, there is a negligible change in the LEE spectrum due to changes of h2, as shown in **Fig. S04**. Thus, different h2 values can produce similar LEE spectra.


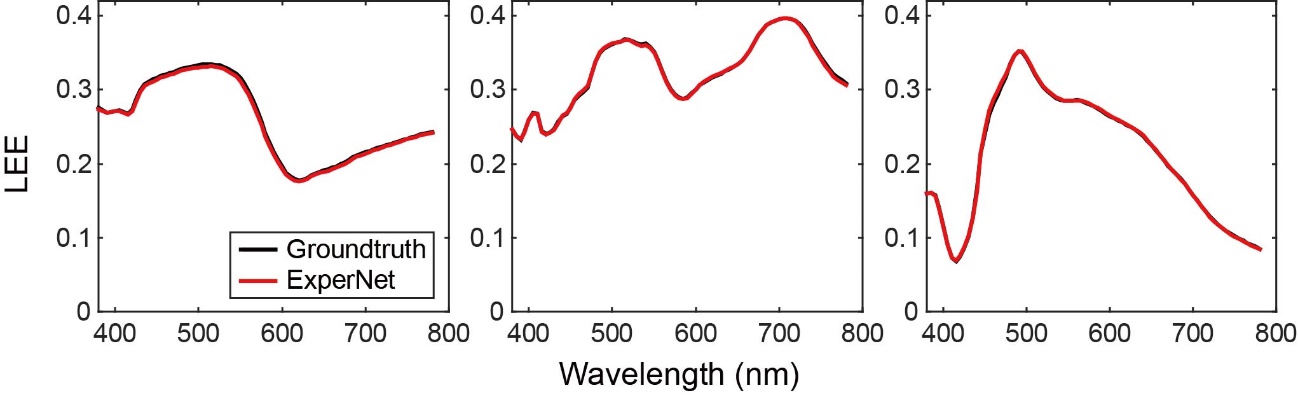


**Supplementary Figure S04.** The comparison of the ground truth LEE spectrum and the LEE spectrum from CPS and predicted input value.

|  |  | h1 (nm) | h2 (nm) | h3 (nm) | h4 (nm) | n1 | n2 |
| --- | --- | --- | --- | --- | --- | --- | --- |
| Struct 01 | G.T | 1120.1 | **37.168** | 226.84 | 45.182 | 1.6137 | 1.5901 |
|  | Predict | 1128.6 | **33.298** | 226.95 | 45.742 | 1.6113 | 1.5927 |
| Struct 02 | G.T | 1248.3 | **34.168** | 244.22 | 341.58 | 1.8761 | 1.9027 |
|  | Predict | 1253.4 | **29.193** | 244.68 | 341.99 | 1.8727 | 1.9029 |
| Struct 03 | G.T | 1301.6 | **25.744** | 36.856 | 104.67 | 1.8782 | 1.7027 |
|  | Predict | 1304.9 | **19.357** | 36.629 | 105.01 | 1.8746 | 1.7014 |

**Supplementary Table S02.** The comparison of the ground truth LEE spectrum and the LEE spectrum from CPS and predicted input value. h2, highlighted structural parameter shows the more than 10% difference between ground truth and predicted value because of its low impact on LEE

**Supplementary section 06**

To evaluate the performance of the systematic error prediction network, we compare it with the results without prediction. When the network does not predict the systematic error, it can provide a baseline for comparison. Supplementary **Fig. S05** shows the normalized network prediction result normalized by the non-prediction result with 1,000 different test samples.

For h1 and h2, the RMSE due to the error prediction network is higher than that without the error prediction network when the number of training samples is small, and it does not improve significantly when the number of training samples increases. This indicates that the network is overfitted due to the small amount of training data. This also leads to a lower network robustness due to the larger output random error.

However, for other network variables (h3, h4, n1, and n3), the network reduces the error of the structural variables by a factor of 0.075-0.4 using a very small amount of training data (less than 100). This shows that transfer learning increases the learning performance while preventing the overfitting. In addition, the network robustness is enhanced by the high accuracy of prediction even with 2% random error.


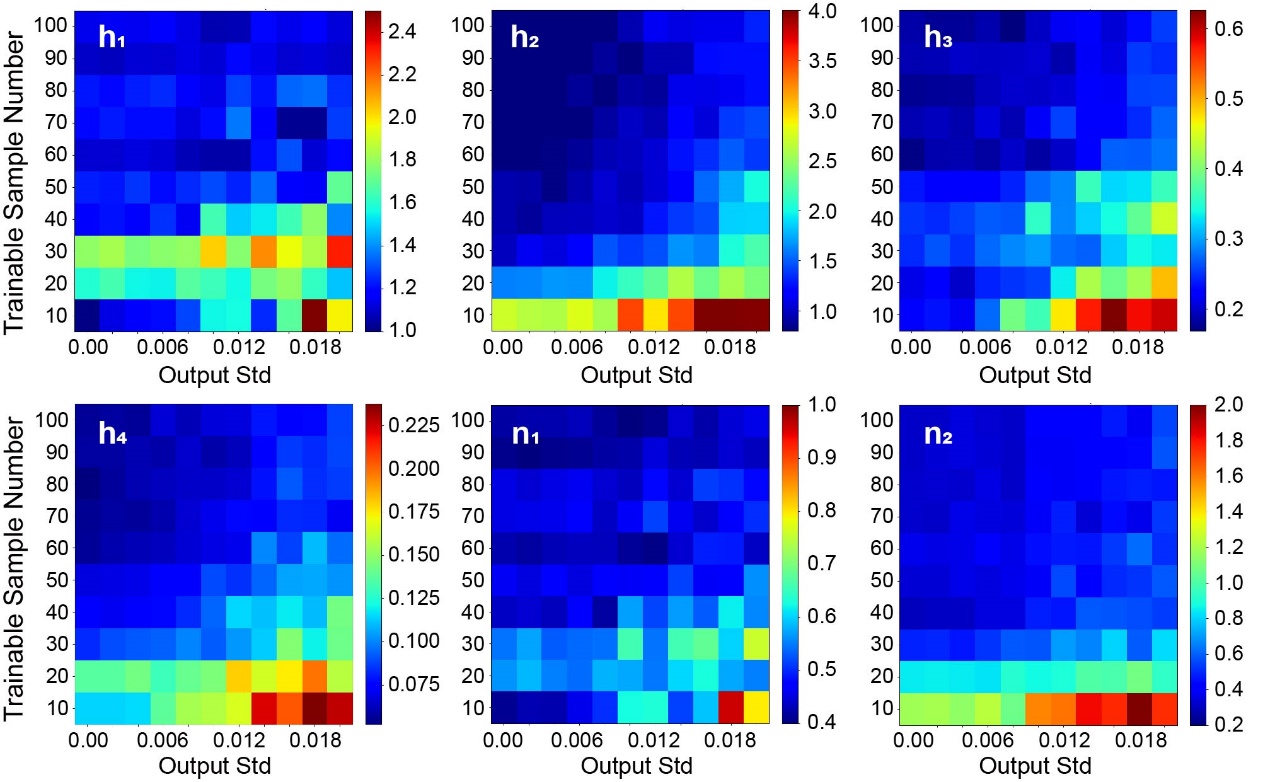


**Supplementary Figure S05.** RMSE of the error prediction network as a function of the number of training sets and random error for parameters h1, h2, h3, h4, n1 and n2.
